# Belief in a just world as a moderator in the face of ageism: a comparative analysis of just world theory and worldview verification theory

**DOI:** 10.3389/fpsyg.2024.1379375

**Published:** 2024-11-08

**Authors:** Eunha Kim

**Affiliations:** Department of Psychology, Ajou University, Suweon, Republic of Korea

**Keywords:** ageism, belief in a just world, just world theory, worldview verification theory, older adults

## Abstract

**Introduction:**

Based on the Just World Theory (JWT) and Worldview Verification Theory (WVT), we conducted two studies to investigate whether a belief in a just world for the self (BJW-self) moderates the relationship between perceived discrimination against older adults (ageism) and self-esteem in a sample of South Koreans older adults.

**Methods:**

In Study 1, we collected survey data from 304 South Koreans aged 65 and older using the scales of perceived ageism, BJW-self, and self-esteem. In Study 2, we randomly assigned 140 South Koreans aged 65 and older to read one of two articles: one describing ageism as pervasive or one describing ageism as rare.

**Results:**

Study 1 revealed that perceived ageism negatively correlated with self-esteem at low levels of BJW-self than at high levels of BJW-self. In Study 2, older adults who were told that ageism is pervasive had lower self-esteem than those who were told that ageism is rare, but this difference was greater for those who rejected BJW-self than for those who endorsed BJW-self.

**Discussion:**

The results support the notion of the JWT that BJW-self mitigates the deleterious effects of perceived ageism on self-esteem.

## 1 Introduction

Ageism refers to bias, prejudice, discriminatory attitudes, or actions towards people based on their age (Butler, 1969). Although ageism can affect any age group, most studies focus on older adults, who are particularly vulnerable to its negative effects on well-being (Kang and Kim, 2022). In South Korea, a society historically influenced by Confucianism and family-oriented values, there has traditionally been an emphasis on hierarchical order and respect for older persons based on age (Kim and Lee, 2020). Consequently, South Koreans have often been assumed to hold more positive attitudes toward older adults. However, recent studies indicate that younger generations are exhibiting higher levels of ageism towards older individuals than previously observed (Kim et al., 2016). More than half of Korean older adults have reported experiencing at least one instance of ageism (Kim et al., 2016).

Increasing levels of ageism in South Korea, driven by demographic shifts and changing social attitudes, have significant implications for older adults’ quality of life. The current population aging and insufficient government support for older persons have exacerbated this issue. For instance, the growing proportion of older adults, coupled with concerns about the financial burden of supporting them, has heightened intergenerational tensions (Kang and Kim, 2022). Additionally, as older workers aged 60 and above increasingly remain in the workforce while younger people face lower employment rates, negative perceptions that older adults are talomg jobs needed by the younger generation have become more prevalent (Kim et al., 2020). Furthermore, Western influences and a shift towards individualism have encouraged younger generations to move away from collective familial obligations.

Globally, ageism has been shown to negatively impact the quality of life of older adults by contributing to social exclusion, diminished self-esteem, and a loss of perceived control over life circumstances (Burnes et al., 2019). Addressing ageism has broad implications for improving the well-being of older adults. As the global population continues to age, ensuring that older adults maintain a high quality of life is increasingly important. Social policies and attitudes that reduce ageism can promote social inclusion, provide better access to health care, and create opportunities for older adults to actively participate in society (Kang and Kim, 2022). By reducing ageism, societies can improve the quality of life for older adults not only in South Korea but also on a global scale.

Aging is a natural stage of life that brings with it several physical, psychological, and social changes. As people age, it is important that they take steps to maintain or improve their quality of life to age actively and healthily. One of the most recommended non-pharmacological strategies for improving quality of life in old age is physical activity (Phillips et al., 2013). Strength training is also an effective intervention. It not only improves muscle strength and balance, but also reduces the frequency and fear of falling, all of which contributes to improving the quality of life and autonomy of older people (Seguin and Nelson, 2003). In addition to exercise, social engagement, networking, and performing activities of daily living have been shown to significantly improve quality of life for older adults (Burnes et al., 2019).

Two key theoretical perspectives—sociometer theory and stereotype embodiment theory—explain how ageism undermines self-esteem and well-being in older adults (Leary, 2005; Levy, 2009). Sociometer theory suggests that self-esteem reflects an individual’s sense of social inclusion and belonging in meaningful relationships (Leary, 2005; Orth et al., 2010). Since ageism signals disapproval, devaluation, and social rejection, it can have a harmful impact on older adults’ self-esteem. Similarly, the stereotype embodiment theory posits that prolonged exposure to ageism leads older adults to internalize negative societal attitudes, which can manifest in self-defeating behaviors and further erode self-esteem (Levy, 2009; Shimizu, 2022).

While studies have reported the negative impact of ageism on self-esteem, such an effect is not always consistent and may vary depending on the circumstances. For example, according to the stress-coping theory, emotional reactions and coping strategies alleviate or exacerbate the psychological burden of perceived discrimination (Miguel et al., 2024; Noh and Kaspar, 2003). Based on this theory, several decades of research have identified protective (e.g., problem-focused coping, social support, and flexible goal adjustment) and risk factors (e.g., suppressive coping and avoidance) (Schmitt et al., 2014; Wei et al., 2008) that mitigate or exacerbate the negative impact of perceived discrimination.

Furthermore, the transactional model, as described by Lazarus and Folkman (1984) assumes that a person’s reaction to discriminatory events is influenced by a two-step cognitive process: primary and secondary appraisal. Initially, an individual evaluates events as either threats or challenges. Following this assessment, they consider their ability to cope with the situations based on the available resources. During these processes, a person’s fundamental assumptions and beliefs about the nature of the world, that is, their worldview, play an important role (Lazarus and Folkman, 1984; Vazquez et al., 2021).

A key aspect of people’s worldviews is the belief that the world is fair, and that people receive the outcomes they deserve [referred to as the belief in a just world (BJW); Rubin and Peplau, 1975]. Before considering the role of BJW in the context of ageism, it is critical to distinguish between the following two constructs of BJW: BJW-self (belief that the world is fair to the self) and BJW-others (belief that the world is fair to others) (Lipkus et al., 1996). Research provides evidence that BJW-self and BJW-others are distinct constructs with different associations. Specifically, BJW-self has been linked to high self-esteem and positive aspects of mental health such as well-being, life satisfaction, and meaning of life (Kim and Jeon, 2019; Sutton and Douglas, 2005; Sutton et al., 2017; Yang et al., 2023). In contrast, BJW-others is related to blaming of and harsh attitudes towards marginalized groups (e.g., people of color, economically disadvantaged, refugees) (Khera et al., 2014). Based on the finding that BJW-self is a better predictor of self-esteem than BJW-others, we primarily examined BJW-self in this study.

Several theories have been used to explain how BJW-self influences an individual’s self-esteem and attribution following an unfair or negative event, such as ageism (Kim and Park, 2018; Lipkus and Siegler, 1993). In particular, the just world theory (JWT) and worldview verification theory (WVT) have received empirical support in this context. Both frameworks propose that the extent to which ageism negatively affects self-esteem depends on the level of the BJW-self; however, they offer two competing hypotheses. According to JWT, high levels of BJW-self are expected to be beneficial for self-esteem by acting as a buffer against the detrimental outcomes of ageism. However, WVT proposes that a strong BJW-self increases the distress of perceived ageism by creating an inconsistency between the worldview (e.g., life is fair) and lived experiences (e.g., ageism) (Lucas et al., 2016).

### 1.1 Just world theory

JWT emphasizes the adaptive function of the BJW-self on psychological well-being (Lambert et al., 1999) as it provides a sense of security and control (Ma et al., 2023). Empirical evidence supports that the BJW-self is linked to positive indicators such as life satisfaction, subjective well-being, self-esteem, and a sense of meaning in life, as well as negative indicators such as depression, anxiety, and insomnia (Hafer and Rubel, 2015; Harding et al., 2020). Because the BJW-self serves such important adaptive functions, individuals have a desire to maintain a strong BJW-self (Lerner and Miller, 1978). Therefore, when individuals experience or observe discrimination that threatens their notion that the world is a just place, they attempt to compensate for it using problem-based coping strategies, such as confrontation and focusing on pursuing goals to reduce injustice (Dalbert, 2001; Liu et al., 2021). If experienced or observed discrimination cannot be compensated for, individuals with high BJW-self reinterpret it to fit their beliefs by downplaying the severity of the experienced or observed injustice, attributing the event to misunderstandings, and interpreting the perpetrator’s action as unintentional or caused by valid reasons unknown to them (Furnham, 2003; Lipkus and Siegler, 1993). As a result, BJW-self has been found to act as a protective factor against the negative effects of injustices and inequalities, such as discrimination and bullying experiences (Correia and Dalbert, 2008; Donat and Wolgast, 2018; Öcel and Aydin, 2010; Sadiq and Bashir, 2015). Therefore, based on JWT, we tested the hypothesis that BJW-self alleviates the negative association between perceived ageism and self-esteem.

### 1.2 Worldview verification theory

WVT offers a different perspective by positing that a strong BJW-self can exacerbate the adverse effects of perceived ageism on self-esteem (Major et al., 2007). The rationale behind this theory is that individuals are motivated to maintain consistency between their beliefs about the world and their experiences, as this consistency helps them predict and navigate their environment. For individuals with a lower BJW-self, perceived ageism aligns with their existing belief that the world is unfair to them. This can paradoxically lead to higher self-esteem. In contrast, for individuals with high levels of BJW-self, perceived discrimination represents a challenge to their beliefs in a just world. Such threats to one’s worldview have been found to increase distress and confusion, which, in turn, lead to lower self-esteem (Townsend et al., 2010).

Previous research supports the theoretical underpinnings of WVT. For example, Major et al. (2007) and Kim and Park (2018) found that women with high BJW had lower self-esteem when experiencing or observing gender discrimination than women with low BJW. In addition, although Townsend et al. (2010) did not directly examine self-esteem, they reported that women with high BJW exhibited higher cardiovascular responses after experiencing gender discrimination than women with low BJW-self. Similarly, Lucas et al. (2016) showed that individuals who endorsed BJW-self experienced higher stress responses when facing discrimination than those who rejected BJW-self. Moreover, other investigations have shown that individuals with high BJW-self are more likely to internalize negative stereotypes or prejudice against their group, leading to lower self-esteem and depression (McCoy and Major, 2007). These findings lend support to WVT by demonstrating that individuals experience adverse outcomes (e.g., decreased low self-esteem and elevated stress levels) when their worldviews (e.g., BJW) and experiences (e.g., perceived ageism) are incongruent.

### 1.3 Current research

**Figure 1:**
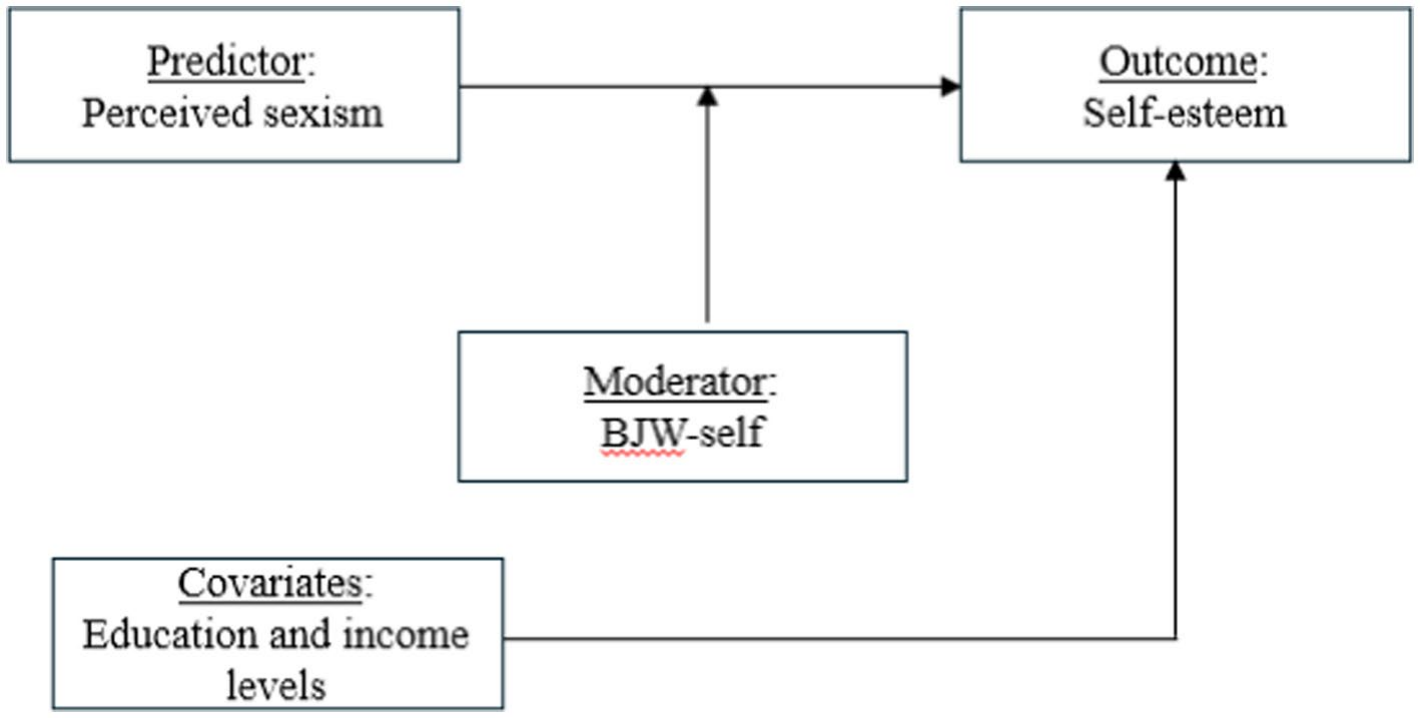
The hypothesized model.

Despite the large amount of research concerning JWT and WVT, to our knowledge, no empirical studies have investigated how the BJW-self acts within the framework of ageism. Therefore, it is unclear whether BJW-self would mitigate or exacerbate the detrimental effects of ageism on self-esteem. Based on JWT and WVT, the present study tested two opposing hypotheses among older South Korean adults. Based on JWT, our first hypothesis was that BJW-self would serve as a protective factor, cushioning the harmful consequences of perceived ageism on self-esteem. Conversely, based on WVT, our second hypothesis was that BJW-self would strengthen the negative association between ageism and self-esteem. Two separate studies were conducted to test these hypotheses. In Study 1, we explored the relationship between perceived ageism and self-esteem as a function of endorsing BJW-self. In Study 2, we analyzed whether endorsing the BJW-self moderated self-esteem in response to narratives portraying ageism as pervasive or rare in South Korea. As shown in Figure 1, we examined the moderating effects of BJW-self on the relationship between perceived ageism and self-esteem, while controlling for the potential effects of education and income levels.

We used both a survey and experiment to address our research questions from different, yet complementary, perspectives. The survey was used to examine the relationships among perceived ageism, BJW-self, and self-esteem. This approach provides insight into how these variables interact in everyday contexts. In contrast, the experiment afforded the opportunity to manipulate key variables, such as controlled exposure to ageism, in order to assess their causal effects on self-esteem. This allows us to test theoretical predictions derived from Just World Theory (JWT) and Worldview Verification Theory (WVT) in a controlled setting. By integrating both methods, we would obtain correlational data through the survey and established causal relationships through the experiment.

## 2 Study 1

### 2.1 Methods

#### 2.1.1 Participants

A total of 304 South Korean adults aged 65 years and older participated in Study 1. The sex ratio was 158 men (52.0%) to 146 women (48.0%) ranging in age from 65 to 79 years (*M* = 71.890, *SD* = 4.015). Most participants were married (*n* = 277, 91.3%). In addition, almost half of the participants had a bachelor’s degree (54.9%, *n* = 167), 28.6% (*n* = 87) had a high school diploma, 13.2% (*n* = 40) had a professional or graduate degree, and 3.3% (*n* = 10) had primary or secondary education. Finally, concerning monthly income, 29.3% (*n* = 89) reported less than $1,150 (2,000,000 Korean won), 38.2% (*n* = 116) reported $1,150 ~ $3,100 (2,000,000 ~ 4,000,000 Korean won), 22.7% (*n* = 69) reported $3,100 ~ $4,600 (4,000,000 ~ 6,000,000 Korean won), 6.3% (*n* = 19) reported $4,600 ~ $6,200 (6,000,000 ~ 8,000,000 Korean won), and 3.6% (*n* = 11) earned more than $6,200 (8,000,000 Korean won). No responses were excluded from the data analysis.

#### 2.1.2 Procedures

The data were collected by MarketLink, a market research company in South Korea. The company sent a recruitment message describing the study’s purpose, procedure, inclusion criteria, and a survey link to potential participants enrolled in their sample pool. At our request, the participants followed the online survey link after providing their written consent. The online survey consisted of scales assessing perceived ageism, BJW-self, and self-esteem. Ethical approval was granted by the Institutional Review Board of Ajou University (IRB number: 202312-HB-004).

#### 2.1.3 Measures

All the measures were administered to the participants in Korean. The scales of BJW-self (Kim et al., 2017) and self-esteem (Schmitt and Allik, 2005) were translated and validated for the Korean sample. The Palmore Ageism Scale, which is not available in Korean, was translated from English to Korean and back-translated for accuracy.

##### 2.1.3.1 Ageism

The Palmore Ageism Survey (Palmore, 2001) was used to assess participants’ encounters with age-based unfair treatment. This scale consists of 20 items that measure experiences of ageism, including prejudice and discrimination. Each item is rated on a 3-point Likert scale, ranging from 0 (“never”) to 2 (“twice or more”), with higher scores indicating more frequent encounters with ageism. The scale has been translated into Korean and has been widely used in South Korean studies to examine the prevalence and effects of ageism. Several studies (Kim, 2012; Lee and Kim, 2016; Lyons et al., 2018) have conducted factor analyses and found that the Korean translated version of this scale effectively captures the construct of ageism in Korea, with its factor structure aligning well with the original conceptual model. Given these findings, it was concluded that no modifications were necessary when applying the scale to the Korean population. The Korean version of the survey has demonstrated satisfactory concurrent and criterion validities. It has also shown high internal consistency, with a reliability coefficient (Cronbach’s alpha) of 0.918, indicating the scale’s robustness in measuring ageism in Korean samples.

##### 2.1.3.2 BJW-self

The Personal BJW Scale (Dalbert, 1999) was used to measure participants’ levels of BJW-self. This scale includes seven items that evaluate beliefs about the fairness of life events. Each item is rated on a 7-point Likert scale from 1 (‘totally disagree’) to 6 (‘totally agree’), with higher scores reflecting a stronger endorsement of BJW-self. The internal consistency reliability of this scale is 0.896. Examples of this scale are “I am usually treated fairly,” “Overall, events in my life are just,” and “I believe that I usually get what I deserve.”

##### 2.1.3.3 Self-esteem

The Self-Esteem Scale (Rosenberg, 1965) was used to measure participants’ levels of self-esteem. This scale includes ten items that assess overall self-regard. Each item is rated on a 3-point Likert scale from 0 (“strongly disagree”) to 4 (“strongly agree”), with higher scores indicating a more positive self-evaluation. The internal consistency reliability of this scale is 0.890. Examples of this scale are “I feel that I have a number of good qualities,” “I am able to do things as well as most other people,” and “On the whole, I am satisfied with myself”.

### 2.2 Results

#### 2.2.1 Preliminary results

As shown in Table 1, the correlations among the study variables were significant. Perceived ageism was negatively correlated with BJW-self and self-esteem, whereas BJW-self was positively correlated with self-esteem. A series of additional correlational analyses, *t*-test, and one-way ANOVA revealed no significant differences in terms of socioeconomic variables (i.e., gender, age, type of work, marital status, and annual income) on perceived ageism, BJW-self, or self-esteem. Despite the lack of significant differences, we included education and income levels as covariates in the main analysis to account for any potential confounding effects.

**Table 1:** Descriptive statistics and correlations among variables.

	1	2	3
1. Perceived ageism	–	−0.361^**^	−0.486^**^
2. BJW-self		–	0.420^**^
3. Self-esteem			–
*M*	24.621	27.029	28.500
SD	8.687	6.389	10.876

^**^p < 0.01.

#### 2.2.2 Main results

**Figure 2:**
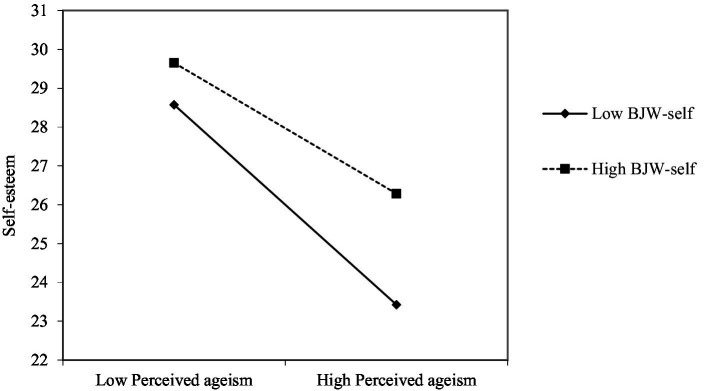
The relationship between perceived ageism and self-esteem at high versus low levels of BJW-self.

The results of the hierarchical multiple regression are reported in Table 2. The interaction between perceived ageism and BJW-self was significant. Moreover, the results supported Hypothesis 1, in that BJW-self mitigated the negative relationship between perceived ageism and self-esteem (Figure 2). More specifically, the negative correlation between perceived ageism and self-esteem were greater at low BJW-self (*β* = −0.372, *t* = −6.664, *p* < 0.001) compared to high BJW-self (β = −0.2340, t = −4.128, *p* < 0.01).

**Table 2:** Analysis of the moderating effect of BJW on the relationship between perceived ageism and self-esteem.

Step		B	SE	*β*	*t*	R^2^
1	Education level	1.040	0.588	0.107	1.829	0.007
	Income level	−0.461	0.389	−0.069	−1.184	
2	Education level	0.186	0.487	0.019	0.382	
	Income level	−0.390	0.330	−0.059	−1.182	
	Perceived ageism	−2.592	0.357	−0.377	−7.258^***^	0.299
	BJW-self	1.972	0.364	0.287	5.412^***^	
3	Education level	0.220	0.485	0.023	0.453	
	Income level	−0.426	0.330	−0.064	−1.293	
	Perceived ageism	−2.574	0.356	−0.374	−0.7.233^**^	0.305
	BJW-self	1.969	0.363	0.286	5.424^***^	
	Perceived ageism X BJW-self	0.551	0.309	0.94	1.863^*^	

^*^p < 0.05, ^***^p < 0.001.

## 3 Study 2

In Study 2, we tested the two hypotheses by manipulating perceived ageism instead of measuring it. Older South Koreans who had been assessed for their BJW-self levels were randomly allocated to read one of two articles: one depicting pervasive discrimination against older people, and one suggesting that such discrimination is rare. According to the JWT, BJW-self correlates with self-esteem and shields against the harmful effects of discrimination. Hence, individuals who reject BJW-self will have lower self-esteem in both situations where they read that discrimination against their group is rare and pervasive compared to individuals who support BJW-self. However, WVT provides evidence that discrimination against one’s group rarely contradicts the beliefs of those with low BJW-self but aligns with the beliefs of those with high BJW-self. Therefore, individuals who reject BJW-self will exhibit lower self-esteem when informed discrimination is rare compared to when it is described as pervasive. The opposite is also expected, that is, those who endorse BJW-self will have lower self-esteem when they read that discrimination is pervasive than when they read that it is rare.

### 3.1 Methods

#### 3.1.1 Participants

A total of 140 South Korean adults aged 65 years and older participated in Study 2. The sample consisted of 64 women (45.7%) and 76 men (54.3%) ranging in age from 65 to 78 years (*M* = 68.450, *SD* = 4.925). Most participants were married (*n* = 129, 92.1%). In addition, nearly half the participants had a bachelor’s degree (50.7%, *n* = 71), 28.6% (*n* = 40) had a high school diploma, 15.7% (*n* = 2) had a professional/graduate degree, and 5.0% (*n* = 7) had elementary or middle school education. Finally, in terms of monthly income, 30.0% (*n* = 42) reported less than $1,150 (2,000,000 Korean won), 40.7% (*n* = 57) reported $1,150 ~ $3,100 (2,000,000 ~ 4,000,000 Korean won), 19.3% (*n* = 27) reported $3,100 ~ $4,600 (4,000,000 ~ 6,000,000 Korean won), 7.1% (*n* = 10) reported $4,600 ~ $6,200 (6,000,000 ~ 8,000,000 Korean won), and 2.9% (*n* = 4) earned more than $6,200 (8,000,000 Korean won). No responses were excluded from the data analysis.

#### 3.1.2 Procedures

Participants were recruited from Seoul, South Korea through posted and online advertisements. After being informed about the study’s aims and procedures and providing their consent, the participants were directed to an online survey. The online survey consisted of scales measuring self-perceptions of aging and BJW-self. Participants were then randomly placed into one of two conditions (pervasive condition, rare condition) and asked to complete their respective online surveys. The assignment was based on the sequence of the participants who contacted the researchers to participate in the study. Half of the participants (*n* = 71) were presented with an article describing discrimination against older individuals as pervasive (pervasive condition). The exact wording of the pervasive discrimination article is as follows:

As you have heard, older adults face pervasive discrimination in many important areas of their lives. Older adults face discrimination and inequality in public transportation, employment, media, politics, and everyday interpersonal interactions. For example, older individuals encounter challenges in the labor market (e.g., being passed over for jobs, promotions, or training opportunities), financial services (e.g., being denied loans or insurance), and public services (e.g., being asked to sit outside in cafés or restaurants). Older adults also face barriers in accessing and using technology, resulting in exclusion from certain services or opportunities that rely on digital platforms, and physical, emotional, or financial abuse by family members, caregivers, or institutions. Recent psychological research has shown that between 80 and 90% of younger adults have negative attitudes toward older people. Younger people generally view older adults as incompetent, irrational, stubborn, resistant to change, and frail. In a survey of Korean people aged 20—30 years last year, more than 80% said that they thought that older people should stay at home and not participate in important tasks.

The other half (*n* = 69) had read an article describing discrimination against older adults as rare (rare condition). The exact wording of these rare conditions is as follows:

As you have heard, discrimination against older adults is becoming less common in many important areas of life. They now face less frequent discrimination and inequality in public transportation, employment, the media, politics, and everyday interpersonal interactions. Many countries have strengthened laws and policies to prevent age-based discrimination, leading to increased job opportunities and financial support for older people. Programs that promote positive interactions between different age groups are also helping to break down stereotypes about older people. Recent psychological research has shown that 80–90% of younger adults have positive attitudes towards older people. Younger people generally view older adults as competent, rational, flexible, adaptable, and strong. In a survey of Korean people aged 20–30 years, last year, more than 80% said that they recognized the significant contributions of older people to economic development and democracy in our society and that they should continue to play an important role as “fellow citizens.”

Following the reading, participants completed measures of the degree to which they believed others viewed their age group favorably (manipulation check) and self-esteem. Upon completing the survey, participants were debriefed with accurate information regarding discrimination against older people and were compensated with $5 for their time. Ethical approval was granted by the Institutional Review Board of the Ajou University (IRB number: 202312-HB-004).

#### 3.1.3 Measures

All measures had previously been translated and validated with Korean samples (Kim et al., 2017; Kim and Park, 2018; Ryu et al., 2012; Schmitt and Allik, 2005).

##### 3.1.3.1 Negative self-perception of aging

The Attitudes towards Own Aging Subscale (Lawton, 1975) of the Philadelphia Geriatric Center Morale Scale (Lawton, 1975) was used to measure participants’ negative views of their aging. This scale includes five items that measure positive and negative perspectives of personal aging. Each item is rated on a 4-point Likert scale ranging from 0 (“strongly disagree”) to 3 (“strongly agree”). To measure negative perceptions of aging, we reversed three items related to positive attitudes. Higher scores reflect a more negative perception of one’s aging. The internal consistency reliability of the scale is 0.784.

##### 3.1.3.2 BJW-self

The Personal BJW Scale (Dalbert, 1999) was used to measure participants’ levels of BJW-self. This scale consists of seven items that assess the belief that the world is fair to the self. Each item is rated on a 7-point Likert scale from 1 (“totally disagree”) to 6 (“totally agree”), with higher scores denoting stronger agreement with BJW-self. The internal consistency reliability of this scale is 0.896.

##### 3.1.3.3 Self-esteem

The Self-Esteem Scale (Rosenberg, 1965) was used to measure participants’ levels of self-esteem. This scale includes ten items that assess overall self-regard. Each item is rated on a 3-point Likert scale from 0 (“strongly disagree”) to 4 (“strongly agree”), with higher scores indicating a more positive self-evaluation. The internal consistency reliability of this scale is 0.890.

##### 3.1.3.4 Manipulation check

The Public Collective Self-esteem subscale of the Collective Self-esteem scale was used to measure participants’ perceptions of how others viewed their age groups. The scale consisted of four items. We changed “social group” in the item to “age group.” The items are “In general, others respect the age group I am a member of;” “Overall, my age group is considered good by others;” “Most people consider my age group, on the average, to be more ineffective than the other age group;” and “In general, others think that the age group I am a member of is unworthy.” To measure the perception that society values one’s age group, we reversed the last two items. Each item is rated on a 4-point Likert scale ranging from 0 (“strongly disagree”) to 3 (“strongly agree”).

### 3.2 Results

#### 3.2.1 Preliminary results

Using *t-*tests, pervasive and rare conditions were compared using pretest measures (i.e., age, negative self-perception of aging, and BJW-self). No significant differences were found (see Table 3). Thus, random assignment to the condition was successful.

**Table 3:** Comparison of the pervasive discrimination and rare discrimination conditions at baseline.

	Pervasive condition		Rare condition		*t*
	*M*	*SD*	*M*	*SD*	
Age	67.449	2.851	67.451	3.013	−0.003
Negative self-perception of aging	7.536	2.246	8.042	2.245	−1.333
BJW-self	26.840	6.060	25.323	5.977	0.149

#### 3.2.2 Manipulation check

The manipulation of perceived discrimination was also successful. We conducted *t-*tests and found significant differences in the public collective self-esteem subscale between the rare and pervasive conditions, with the rare condition showing higher scores than the pervasive condition (*t* = 2.552, *p* < 0.01). Moreover, according to a hierarchical regression analysis with the condition (rare condition = 0, pervasive condition = 1) as a dichotomous variable and BJW-self as a continuous variable in Step 1, the condition significantly affected public collective self-esteem (*β* = −0.38, *p* < 0.01). In addition, BJW-self was significantly related to public collective self-esteem (β = 0.28, *p* < 0.05). However, the addition of the Condition X BJW-self interaction term to Step 2 was not significant.

#### 3.2.3 Main results

**Figure 3:**
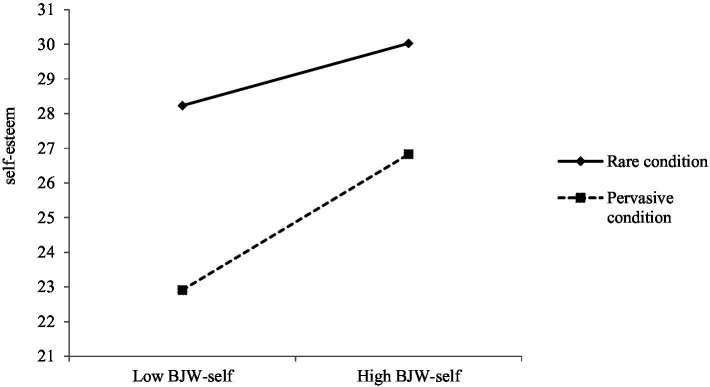
The relationship between BJW-self and self-esteem in the rare discrimination condition and the pervasive discrimination condition.

To test our hypotheses regarding how BJW-self influences the relationship between perceived discrimination and self-esteem, we conducted a hierarchical multiple regression using IBM SPSS ver.25.0. The first step involved entering the main effects of BJW-self and condition. Then, the interaction term between Condition and BJW-self was added in Step 2. The results, detailed in Table 4, revealed significant main effects for BJW-self and the condition in Step 1. In addition, the interaction between the Condition and BJW-self proved to be significant in Step 2. As shown in Figure 3, examination of the simple slopes revealed that, in line with JWT expectations, BJW-self was positively and significantly correlated with self-esteem in the pervasive condition (β = 0.38, *p* < 0.01). In addition, BJW-self was positively associated with self-esteem, but this relationship was only marginal (β = 0.17, *p* = 0.07) in the rare condition. Thus, a high BJW-self is positively related to self-esteem in the rare and pervasive conditions, but this positive relationship is stronger in the pervasive condition than in the rare condition. These results are consistent with the JWT finding that learning that prejudice is pervasive is less harmful for those with high BJW-self than for those with low BJW-self.

**Table 4:** Analysis of the moderating effect of BJW-self on the relationship between perceived ageism and self-esteem.

Step		B	SE	*β*	*t*	R^2^
1	Condition	−0.2.121	0.888	−0.190	−2.389^**^	0.143^* **^
	BJW-self	1.785	0.446	0.318	4.006	
2	Condition	−2.139	0.890	−0.190	−2.394^**^	0.189^* *^
	BJW	1.428	0.656	0.254	2.178	
	Condition X BJW-self	0.665	0.073	0.531	1.781^*^	

^*^p < 0.05; ^**^ *p* < 0.01.

## 4 General discussion

### 4.1 Insights and implications

Across the two studies, we found that perceived ageism, whether measured as an individual difference variable or manipulated experimentally, was negatively associated with self-esteem overall (see also Garstka et al., 2004; Kim, 2020, for similar findings). This finding is consistent with theories suggesting that experiencing a devaluation of one’s social identity leads to lower self-esteem (e.g., the stereotype embodiment theory). However, our results suggest that perceived ageism does not equally affect all participants. Perceived ageism was found to have fewer adverse effects on self-esteem among participants who strongly endorsed BJW-self than among those who rejected it. This pattern supports JWT, suggesting that a strong BJW-self is associated with the denial of existing inequalities and thus buffers the potential stress of perceived discrimination on self-esteem (Bastounis and Minibas-Poussard, 2012). In addition, our findings confirm Dalbert’s (1999) assertion that the BJW-self is the main resource that protects marginalized individuals from the negative effects of unfair treatment by enabling them to adaptively cope with these events and recognize positive qualities in the world.

Although no studies have examined the role of BJW-self in the context of ageism, we can infer several mechanisms for how BJW-self helps the older adults maintain high self-esteem. First, when confronted with ageism, either observed or experienced, older adults high in the BJW-self tend to evaluate the situation to restore justice in several ways (Dalbert, 1999; Kim and Kwon, 2024). For example, individuals with high BJW-self attempt to interpret observed or experienced unfairness as an exception and minimize its consequences. In addition, they try to justify unfairness as partially self-inflicted and avoid self-focused rumination (Lucas et al., 2010). These cognitive styles lead to a reduction in negative emotions caused by discrimination (e.g., anger) and help maintain self-esteem (Dalbert, 2002).

Second, when experiencing ageism, older individuals high in BJW-self are likely to take positive actions (e.g., advocating for their rights or engaging in activities that promote positive aging) rather than succumbing to feelings of victimization. This is possible because individuals with high BJW-self are confident that their efforts will lead to positive outcomes (Ucar et al., 2019). In addition, rich literature links the BJW-self to positive future expectations and hopes (Correia et al., 2023; Xie et al., 2011). Third, because BJW-self is related to interpersonal trust (Guo et al., 2022), it empowers older individuals to reach out to others for assistance and support when facing ageism, thereby strengthening the positive social connections that contribute to high self-esteem.

However, it should be noted that our results contradict the prediction of WVT or empirical findings that support WVT. According to WVT, perceived discrimination against one’s group is negatively associated with self-esteem among individuals who endorse BJW-self, whereas it is positively associated with self-esteem among individuals who reject BJW-self. Studies have provided evidence for WVT in the context of discrimination based on ethnic groups and gender (Lucas et al., 2016; Major et al., 2007). The divergence of the results of this study from previous findings can be attributed to the age of the participants and features of ageism.

More specifically, unlike most previous studies that focused on college students or younger adults (e.g., Lucas et al., 2016; Major et al., 2007; Kim and Park, 2018), our participants were older individuals aged 65 and older. Older individuals form their beliefs about the world based on diverse experiences over a long period, which means that their BJW-self tends to be more stable than that of younger adults. As a result, even when confronted with situations (e.g., discrimination) that are inconsistent with their beliefs (i.e., BJW-self), older individuals high in BJW-self are less likely to experience confusion and uncertainty. Thus, observed or perceived ageism does not threaten the sense of worth or purpose. In addition, the less time people have left in their lives, the more they are compelled to find meaning in their lives (Dzuka and Dalbert, 2006). BJW-self provides a framework for interpreting unfair treatment in a meaningful way, as people high in BJW-self tend to ruminate less about negative events (Correia et al., 2023; Lucas et al., 2010).

In addition, unlike racism and sexism, which are experienced from childhood, ageism is experienced later in life. It is reasonable to assume that older adults high in BJW-self are likely to have experienced little discrimination in their youth. Moreover, given that a safe and trusting environment is essential for the development of the BJW-self (Nudelman, 2013), older individuals high in BJW-self have probably experienced positive relationships with others. Owing to such experiences of rare discrimination and positive interpersonal relationships in the past, older people with high BJW-self are likely to be confident that they will be treated fairly by others. Although ageism is inconsistent with their BJW-self, such confidence allows older people high in BJW to rationalize their beliefs (e.g., by playing down the perpetrator’s actions, perceiving them as unintentional, or minimizing the situation) (Dalbert, 1999).

Another reason our findings contradict WVT is collectivism. While the participants in relevant studies (e.g., Lucas et al., 2016; Major et al., 2007) were from individualistic cultures, our participants were strongly influenced by collectivism. In Kim and Park (2018) study, the participants were South Korean; however, they were college students who were more likely to endorse individualism. Research has revealed that people who are high in collectivism tend to rationalize their BJW-self, even when they encounter injustice (e.g., their efforts are not rewarded), than people who are high in individualism (Yang et al., 2022). This pattern of collectivism is related to high motivation to seek harmony with others and avoid social exclusion (Cheung et al., 2011).

By identifying a cognitive factor (BJW-self) related to older adults’ experiences of ageism, our study provides several practical implications for counseling older adults. For example, when working with older clients, clinicians are encouraged to explore their experiences of ageism and how these experiences affect self-esteem. In doing so, it is essential to keep in mind that clients with low BJW-self are particularly vulnerable to low self-esteem when confronted with ageism. Considering our findings, clinicians also should be encouraged to help clients develop positive perceptions of themselves by exploring their biases towards older groups of people and identifying their accomplishments. Moreover, clients low in BJW-self will benefit from reflecting on past events that contributed to the formation of BJW-self and examine whether thinking patterns such as black-and-white thinking, overgeneralization, and mental filters were present. Encouraging clients to evaluate their thoughts and discover alternative perspectives is crucial. In addition, it would be important to explore lived examples of valuing the experiences and contributions of older adults to society.

In contrast, when working with older clients high in BJW-self, clinicians should be aware that although BJW-self allows them to downplay or adaptively cope with ageism, it is possible that their attempts to restore justice may not always work. Therefore, it is necessary for clinicians to discuss the reality of ageism (e.g., negative perceptions of aging may be prevalent) and alternative responses to it (e.g., developing a positive self-perception of aging and seeking support) with their clients. In addition, when working with older clients high in BJW-self, it is important for clinicians to be aware that an overly rigid BJW-self can have negative consequences, such as self-blame. Therefore, while BJW-self can offer benefits in coping with ageism, it is crucial to promote a balanced view that recognizes systemic injustice and the complex nature of discrimination.

### 4.2 Limitations and suggestions for future research

Several limitations should be considered when interpreting our findings. First, the cross-sectional nature of the data prevented us from making casual inferences. Moreover, our reliance on self-reported measures presents its own set of limitations. To address tis limitation, we manipulated perceived discrimination experimentally, but measured BJW-self and self-esteem through self-reported measures. Third, we did not control for self-perceptions of aging (e.g., internalized ageism). While existing research indicates a persistent link between perceived discrimination and self-esteem, after controlling for internalized ageism (Bae and Kim, 2022), our findings indicate that internalized ageism is related to self-esteem (Bergman, 2022; Orth et al., 2010), which should be considered in future research. Fourth, a limitation of our study is that the manipulation of perceived ageism in Study 2 may have inadvertently influenced other factors, such as emotional valence and attitudes toward aging and older adults. For instance, participants in the pervasive condition may have experienced more negative feelings and developed more negative attitudes compared to those in the rare condition. Although our manipulation aimed to specifically alter levels of perceived ageism, these additional factors could confound the results. Finally, using a short essay to influence perceptions of ageism in Study 2 has limitations. Self-esteem and perceived discrimination among older adults are likely to change over time. Therefore, future research is necessary to examine the long-term effects of perceived ageism on self-esteem through repeated exposure to information about ageism or exploring participants’ lived experiences. Notwithstanding these limitations, our findings offer valuable insights into the immediate effects of perceived ageism and show that BJW-self plays a role in this relationship.

Finally, although the findings from Study 1 and Study 2 were largely comparable, supporting the role of BJW-self in moderating the relationship between perceived ageism and self-esteem, it is crucial to highlight the qualitative differences between the two studies, particularly regarding the nature of ageism exposure. Study 1 focused on participants’ personal experiences with ageism, whereas Study 2 employed hypothetical scenarios to prime participants. As Study 2 did not target direct personal experiences of ageism, it is possible that participants’ responses to the priming task may have varied based on individual factors, such as subjective age, socioeconomic status, or the personal relevance of the scenarios. For example, participants who do not perceive themselves as “old” or who have not directly experienced ageism due to socioeconomic privilege may have found it challenging to relate to the pervasive ageism scenario. It would be beneficial for future studies to aim to replicate our findings by priming participants with direct experiences of ageism and further exploring how factors such as age, socioeconomic status, and the frequency of ageism exposure interact with BJW-self in shaping responses to both personal and indirect experiences of ageism.

## 5 Conclusion

Although there is growing evidence of the role of BJW-self in the relationship between perceived discrimination and self-esteem, our study is the first attempt to explore this issue in the context of ageism. The results of Study 1 indicated that perceived ageism was negatively correlated with self-esteem, particularly among individuals with low levels of BJW-self. In Study 2, older adults who were informed that ageism is pervasive exhibited lower levels of self-esteem than those who were informed that ageism is rare. However, this discrepancy in self-esteem was more pronounced among those with lower BJW-self than among those who endorsed it. Our findings indicate that the impact of perceived ageism on self-esteem is not uniform; those with stronger BJW-self experience fewer negative effects on self-esteem compared to those who did not endorse this belief.

One of the key strengths of this study, compared to similar research, is its focus on older adults—a group often underrepresented in studies on discrimination. While many studies examine younger individuals, our study provides unique insights into how older adults process and cope with ageism, which often becomes more salient later in life. In addition, our study holds important theorical implications. It advances the literature on stereotype embodiment theory and JWT demonstrating that BJW-self moderates the effects of ageism on self-esteem. Our findings underscore the stability of BJW-self in older adults and its role in fostering adaptive coping mechanisms in response to age-related discrimination.

Practically, these findings have significant implications for improving the quality of life for older adults. Clinicians working with clients low in BJW-self should focus on exploring past experiences that may have shaped these beliefs, identifying cognitive distortions such as black-and-white thinking or overgeneralization, and helping clients develop alternative perspectives. For individuals high in BJW-self, interventions should aim to balance the protective benefits of this belief with a recognition of the systemic nature of ageism, ensuring that clients can cope with the injustices they may face without resorting to self-blame.

However, this study is not without limitations. First, the sample was limited to older adults aged 65 and above, which limits the generalizability of the findings to younger populations or individuals experiencing other forms of discrimination. Additionally, while this study sheds light on the role of BJW-self, it does not fully examine the long-term effects of this belief system, particularly whether overly rigid beliefs could have detrimental effects in other areas of life. Future research should address these limitations by examining the role of BJW-self across different age groups and types of discrimination and by conducting longitudinal studies to track the stability and potential long-term effects of BJW-self over time.

## Data Availability

The raw data supporting the conclusions of this article will be made available by the authors, without undue reservation.
